# Late cardiopulmonary morbidity in patients with Hodgkin lymphoma: a retrospective nationwide registry study

**DOI:** 10.1007/s00277-026-07193-9

**Published:** 2026-07-22

**Authors:** Noora Hannuksela, Outi Kuittinen, Aino Rönkä, Anna Anttalainen, Liisa Ukkola-Vuoti, Iiro Toppila, Johanna Vikkula, Jan Seppälä, Kristiina Vuolukka, Katja Marin, Hanne Kuitunen, Tatu Miettinen, Anu Partanen

**Affiliations:** 1 Oncology, Faculty of Health Medicine, School of Medicine, Institute of Clinical Medicine, University of Eastern Finland, Kuopio, Finland; 2 Department of Oncology, Kuopio University Hospital, Kuopio, Finland; 3 Medaffcon Oy, Espoo, Finland; 4 Department of Oncology, Department of Radiotherapy, Kuopio University Hospital, Kuopio, Finland; 5 Cancer Centre, Oulu University Hospital, Oulu, Finland; 6 Takeda Oy, Helsinki, Finland; 7 Department of Medicine, Kuopio University Hospital, Kuopio, Finland

**Keywords:** Hodgkin lymphoma, Late toxicity, Late morbidity, Cardiovascular comorbidity, Pulmonary comorbidity, Radiotherapy

## Abstract

Hodgkin lymphoma (HL) is a B-cell neoplasm with a cure rate of 90%. Previous data on late morbidity in patients with HL cover the era of older treatment modalities. This real-world study consisted of 2912 patients diagnosed with HL between 2000 and 2019 in Finland and aimed to clarify the incidence of late-onset cardiopulmonary morbidity compared to matched controls. Patient data were collected from four nationwide Finnish healthcare registries. We reported a higher occurrence of coronary artery disease in patients aged < 50 years at diagnosis than in controls (RR 3.80 (2.16–6.74), *p* < 0001). Additionally, a higher incidence of both heart failure (RR 1.68 (1.28–2.21), *p* < 0.001) and valvular heart disease (RR 1.80 (1.17–2.78), *p* = 0.006) was detected in patients aged ≥ 50 years at diagnosis than in the controls. Remarkably, in the < 50 years age group, the incidences of pneumonia (RR 4.89 (3.58–6.71), *p* < 0.001) and pulmonary embolism (RR 7.78 (2.75–21.95), *p* < 0.001) were higher than those in the control group. In conclusion, with the current treatment modalities, late cardiopulmonary morbidity in long-term survivors remains a great challenge. A strategy for primary prevention and early detection of late toxicity remains crucial and a critical evaluation of current treatment practices is warranted.

**Supplementary Information:**

The online version contains supplementary material available at https://doi.org/10.1007/s00277-026-07193-9.

## Introduction

Hodgkin lymphoma (HL) is a malignant B-cell neoplasm [1, 2] classified as classical HL (cHL) or nodular lymphocyte-predominant HL (NLPHL) [3]. The incidence of HL has a bimodal distribution pattern in patients aged 20–35 and 60–75 years [4, 5]. Combined modality treatment (CMT) with chemotherapy ± involved site radiotherapy (ISRT) [3, 6–8] has resulted in a cure rate of up to 90% [3, 9].

Despite good disease control, late morbidity, especially among younger long-term survivors of HL, remains challenging [10, 11]. Cumulative doses of anthracycline-containing chemotherapies may increase the risk for cardiovascular diseases by inducing oxidative stress or myocyte apoptosis [10, 12, 13]. The EORTC-LYSA trials, including patients with HL treated between 1964 and 2004, reported that after a median follow-up of 9 years from the initial treatment, 37% of patients with HL were diagnosed with cardiovascular disease, 19% had ischaemic heart disease, 12% had congestive heart failure (CHF), and 11% had valvular heart disease [14]. A higher risk of heart disease may persist decades after HL treatment [15].

Due to the limited amount of bleomycin hydrolase in the lungs, bleomycin may cause drug-induced pneumonitis, especially in the elderly population [16]. In addition, RT may induce radiation pneumonitis and pulmonary fibrosis [17, 18]. A previous study observed excess visits to physicians due to infections in patients before the HL diagnosis compared with controls [19]. In addition, a previous Danish registry study of children observed a 60-fold standardised incidence ratio for HL diagnosis within 6 months of a hospital-based diagnosis of pneumonia [20]. In contrast, the US SEER database suggested no statistically significant association between HL and pneumonia or bronchitis [21].

Earlier research on the risk analysis of late morbidity after HL treatment was based on older treatment modalities, which included more toxic MOPP (mechlorethamine, vincristine, procarbazine, and prednisone) or MOPP-ABVD (mechlorethamine, vincristine, procarbazine, prednisone, doxorubicin, bleomycin, vinblastine, and dacarbazine) chemotherapy regimens and large-field mantle radiotherapy with higher radiation doses [15, 22]. Publications on cardiopulmonary morbidity after treatment according to the current guidelines are scarce [23]. This real-world study aimed to clarify the incidence of late-onset cardiopulmonary morbidity and its association with radiotherapy in patients diagnosed with HL in Finland between 2000 and 2019.

## Methods

### Data collection

This retrospective study was based on nationwide Finnish healthcare registries, which account for the entire Finnish population (approximately 5.5 million people in 2020). We used four different sources for data collection: the Finnish Cancer Registry (FCR), the National Institute of Health and Welfare (THL), Statistics Finland, and the Digital and Population Data Services Agency (DPA). Patients diagnosed for the first time with HL between 2000 and 2019 and without pre-existing non-HL diagnosis were included (*n* = 2912) based on FCR records. Patients were individually matched with controls by age, sex, and home municipality in Finland in a 1:1 ratio using DPA records, thus forming the control group. Controls were eligible for the study if they were alive at the time of HL diagnosis of the corresponding case and had no lymphoma diagnoses in FCR at any time. The baseline comorbidities (diabetes, hypertension, and dyslipidaemia) were collected from three years prior to HL diagnosis. Follow-up was started at the time of diagnosis (of the corresponding HL patient) and was continued until 31.12.2019 or death, whichever occurred first. A flowchart of the data collection is presented in Figure S1.

### Ethics

This study adhered to the ethical principles of the Declaration of Helsinki and was conducted in accordance with the Good Clinical Practice Statement. Patient records were analyzed in a secure environment and the data used were pseudonymized, resulting in a low risk of compromised data security. The data collection process was approved by the Finnish Social and Health Data Permit Authority (Findata, permit no. THL/1541/14.02.00/2021) and Statistics Finland (permit no. TK/3616/07.03.00/2021).

### Statistics

Statistical analyses were conducted using R (version 4.0.3) and stratified according to age and radiation status (received radiotherapy within year from HL diagnosis; control groups assigned based on the RT status of the corresponding case). The cutoff point of the age groups (50 years at diagnosis) was based on previous findings of changes in incidence [24] and relative survival [25]. Demographic characteristics were assessed at the time of diagnosis. Stem cell transplant status (SCT) was assessed throughout the follow-up and radiotherapy (RT) during the first year after HL diagnosis. RT and SCT were assessed only in HL patients. Comorbidities (Table S1) were determined using speciality care registry diagnoses (available 1997–2019, providing at least 3 years of baseline data for all patients and controls) alone, as primary care data were available only from 2011 onwards. The Charlson comorbidity index (CCI) was calculated at baseline, excluding lymphomas (ICD10 C81-C85) [26].

The studied comorbidities and corresponding ICD10-codes are presented in Table S1. The occurrence of comorbidities over the follow-up time was examined with multi-state Aalen-Johanssen estimators, accounting for the competing risk of death. Comorbidity onset was defined as the date of the first recorded diagnosis. Comorbidities identified within the three years prior to the index date were regarded as baseline and excluded from incidence calculations during follow-up. Cumulative incidences of comorbidities diagnosed after the initial HL diagnosis are reported. Differences in comorbidity incidence rates between cases and controls were analyzed with Poisson regression, with logarithm follow-up length durations included as an offset. This analysis was conducted separately for the comorbidities diagnosed during baseline period and follow-up. Rate ratios (RR) at 3-year baseline and during follow-up are reported to quantify these differences. Factors associated with the occurrence of comorbidities were studied with Cox proportional hazards models, with death and end of follow-up treated as censoring events. Controls and patients with baseline comorbidities were excluded from the analysis. For continuous variables, age and calendar year of diagnosis, HR represents the average effect per one-year increase. Radiotherapy and stem cell transplantation (SCT) were time-dependent covariates. Proportional hazard assumptions were checked. All statistical tests were two-sided unless otherwise informed; no paired testing was applied due to small sample sizes in some groups. Results and *p*-values were descriptive, with no multiple testing correction. All analyses used a significance level of 0.05. Patient numbers below 5 were censored to maintain data privacy.

## Results

### Patient characteristics

Our nationwide registry study consisted of 2912 patients diagnosed with HL (ICD-10: C81*) between 1.1.2000 and 31.12.2019 in Finland. Median observed follow-up was 9.2 (IQR 4.2, 14.2) years for cases and 9.6 (4.7, 14.6) years for controls in the younger patients, and respectively 3.7 (0.8, 8.4) and 6.1 (2.9, 10.8) years for the older group. The patient characteristics according to age are presented in Table 1.

**Table 1:** Characteristics and demographics of HL patients and controls according to two age groups

Variable		Younger (< 50) cohort			Older (≥ 50) cohort		
		Case *n* = 1855 (%)	Control *n* = 1855 (%)	*P*-value	Case *n* = 1057 (%)	Control *n* = 1057 (%)	*P*-value
Age, years, median [IQR]		28.87 [20.65,35.71]	28.87 [20.65,35.71]		66.21 [58.89,75.22]	66.21 [58.89,75.22]	
HL subtype, n (%)	cHL	1650 (88.9)	-		860 (81.4)	-	
	NLPHL	205 (11.1)	-		197 (18.6)	-	
Sex, n (%)	Male	994 (53.6)	994 (53.6)		661 (62.5)	661 (62.5)	
	Female	861 (46.4)	861 (46.1)		396 (37.5)	396 (37.5)	
CCI, n (%)	0	1698 (91.5)	1792 (96.6)	< 0.001	706 (66.8)	859 (81.3)	< 0.001
	1–2	127 (6.8)	63 (3.4)		240 (22.7)	159 (15.0)	
	3+	36 (1.6)	0		111 (10.5)	39 (3.7)	
Baseline comorbidities, n (%)	Diabetes (ICD-10: E10-E11)	14 (0.8)	19 (1.0)	0.484	95 (9.0)	51 (4.8)	< 0.001
	Hypertension (ICD-10: I10)	13 (0.7)	< 5	0.052	171 (16.2)	101 (9.6)	< 0.001
	Dyslipidaemia (ICD-10: E78)	< 5	0	0.479	15 (1.4)	10 (0.9)	0.421
Treatment							
Radiotherapy within 1 year		539 (29.1)	-		253 (23.9)	-	
Transplantation	No SCT	1710 (92.2)			> 1015 (> 96.0)	-	
	Autologous	108 (5.8)	-		32 (3.0)		
	Allogenic	16 (0.9)	-		< 5 (< 0.5)	-	
	Allogenic & autologous	21 (1.1)	-		5 (0.5)	-	

*IQR* interquartile range, *CCI* Charlson comorbidity index, *cHL* classical Hodgkin lymphoma, *NLPHL* nodular lymphocyte-predominant Hodgkin lymphoma

### Cardiovascular diseases

#### Coronary artery disease or infarction

The incidence rate of coronary artery disease (CAD) in the younger patient group was higher than in controls (RR 3.80 (2.16–6.74), *p* < 0001) (Table 2A, Figure S2) and older age at diagnosis was associated with a higher risk (HR 1.06, 95% CI 1.03–1.09, *p* < 0.05) (Table 3A). In the older group, the incidence of CAD was comparable between patients and controls. Older age at diagnosis was associated with a higher risk (HR 1.07, 95% CI 1.05–1.10, *p* < 0.05) and the female sex with a significantly lower risk (HR 0.44, 95% CI 0.29–0.67, *p* < 0.05) (Table 3B) for CAD.

**Table 2:** Cumulative incidence (%) of newly diagnosed cardiovascular diseases and corresponding rate ratios (RR) in the patients with HL

	Group	Follow-up time (years)								Baseline RR, p-value	Follow-up RR, p-value
		0	1		5	10	15	20			
A) in the younger (< 50 years) group											
Cum. incidence, (%) coronary artery disease or infraction	*HL*	0	0.3		1.2	3.0	4.9	7.3		2.00 (0.36–10.97), *p* = 0.423	3.80 (2.16–6.74), *p* < 0.001
	*control*	0	0		0.4	0.8	1.2	2.3			
Cum. incidence, (%) arrhythmia	*HL*	0	0.3		0.6	1.0	2.1	3.7		0.67 (0.11–3.95), *p* = 0.657	2.25 (1.13–4.46), *p* = 0.020
	*control*	0	0		0.4	0.7	0.9	1.4			
Cum. incidence, (%) valvular heart disease	*HL*	0	0.3		0.8	1.5	2.1	2.8		2.5 (0.48–13.02), *p* = 0.273	†
	*control*	0	0		0.1	0.1	0.7	0.7			
Cum. incidence, (%) cardiomyopathy	*HL*	0	0		0.1	0.4	0.8	1.4		†	2.07 (0.71–6.1) *p* = 0.183
	*control*	0	0		0	0.4	1.0	1.0			
Cum. incidence, (%) congestive heart failure	*HL*	0	0.1		0.5	0.8	1.8	2.8		†	2.72 (1.19–6.19), *p* = 0.016
	*control*	0	0		0	0.4	1.0	1.0			
B) In the older (≥ 50 years) group											
Cum. incidence, (%) coronary artery disease or infarction	*HL*	0		4.0	8.6	12.8	17.3		20.9	1.52 (1.11–2.08), *p* = 0.009	1.27 (0.99–1.64), *p* = 0.062
	*control*	0		2.7	9.0	14.9	19.3		22.2		
Cum. incidence, (%) arrhythmia	*HL*	0		2.9	7.8	11.6	14.2		15.5	1.15 (0.84–1.57), *p* = 0.374	1.11 (0.86–1.43), *p* = 0.441
	*control*	0		1.3	8.4	13.8	19.6		26.1		
Cum. incidence, (%) valvular heart disease	*HL*	0		1.3	3.4	5.2	7.0		10.6	0.95 (0.52–1.75), *p* = 0.876	1.8 (1.17–2.78), *p* = 0.006
	*control*	0		0.6	2.4	4.0	5.4		7.3		
Cum. incidence, (%) cardiomyopathy	*HL*	0		0.4	1.3	2.6	2.8		5.8	1.22 (0.51–2.95) *p* = 0.655	3.35 (1.53–7.34) *p* = 0.002
	*control*	0		0	0.6	1.2	1.2		2.4		
Cum. incidence, (%) congestive heart failure	*HL*	0		2.9	8.0	12.9	16.2		20.1	2.04 (1.25–3.32), *p* = 0.005	1.68 (1.28–2.21), *p* < 0.001
	*control*	0		1.5	5.3	10.9	16.6		23.5		

† Not available due to zero events in cases and/or controls*RR* rate ratio

#### Atrial arrhythmia

In the younger group, the occurrence of arrhythmia was higher in patients with HL than in controls (RR 2.25 (1.13–4.46), *p* = 0.020) (Table 2A). A higher CCI and older age at diagnosis were associated with a higher risk (HR 1.37, 95% CI 1.11–1.68, *p* = 0.0280 and HR 1.05, 95% CI 1.05–1.09, *p* = 0.0170, respectively) (Table 3A) and female sex with lower risk of arrhythmia (HR 0.39, 95% CI 0.16-1.00, *p* = 0.0493). In addition, radiotherapy in the younger patients was associated with a fourfold risk of arrhythmia (HR 4.05, 95% CI 1.62–10.14, *p* = 0.0028) (Table S2).

In the older group, older age at HL diagnosis was associated with arrhythmia (HR 1.10, 95% CI 1.07–1.12, *p* < 0.001) (Table 3B), whereas the incidence rate was comparable between patients and controls (RR 1.11 (0.86–1.43), *p* = 0.441).

#### Valvular heart disease

The incidence of valvular problems did not differ between younger patients and controls (Table 2A) whereas the incidence rate was higher in older patients than in controls (RR 1.80 (1.17–2.78), *p* = 0.006) (Table 2B, Figure S3). Older age at diagnosis (HR 1.07, 95% CI 1.03–1.1, *p* < 0.001), and higher CCI at baseline (HR 1.27, 95% CI 1.09–1.49, *p* = 0.0026) were associated with an elevated risk for valvular disease in this older group (Table 3B).

#### Cardiomyopathy and congestive heart failure

The incidence of cardiomyopathy was low in the younger group (Table 2A). The older age at diagnosis (HR 1.07, 95% CI 1.0-1.14, *p* = 0.0352) and higher CCI at baseline (HR 1.40, 95% CI 1.05–1.87, *p* = 0.0209) was associated with an increased risk for cardiomyopathy (Table 3A). The incidence rate of cardiomyopathy was higher in the elderly group without radiotherapy than in the control group (RR 3.31 (1.46–7.56), *p* = 0.005) (Table S2). Female sex was associated with a significantly lower risk for cardiomyopathy (HR 0.29, 95% CI 0.09–0.87, *p* = 0.0277) (Table 3B).

The incidence rate of congestive heart failure (CHF) was 2.72-fold higher in younger patients than in controls (*p* = 0.016) (Table 2A). Older age at diagnosis (HR 1.05, 95% CI 1.01–1.10, *p* = 0.016) and higher CCI at baseline (HR 1.45, 95% CI 1.19–1.77, *p* < 0.001) were associated with CHF (Table 3A). In the older group, the incidence rate of CHF at baseline (RR 2.04 (1.25–3.32), *p* = 0.005) and during follow-up (RR 1.68 (1.28–2.21), *p* < 0.001) was higher in patients than in controls (Table 2B). The older age at diagnosis (HR 1.11, 95% CI 1.08–1.13, *p* < 0.001) was linked with an increased risk, whereas the NLPHL subtype (HR 0.54, 95% CI 0.30–0.94, *p* = 0.030) was associated with a lower risk of CHF in those patients (Table 3B).

**Table 3:** Cox multivariable model fit for hazard for newly diagnosed CVDs in patients with HL

			HR, (95% CI),*p*-value						
Covariate			Coronary artery disease or infarction	Atrial arrhythmia		Valvular heart disease		Cardiomyopathy	Congestive heart failure
A) in the younger (< 50 years) group									
Age at diagnosis			1.06 (1.03–1.09),*p* < 0.001	1.05 (1.01–1.09),*p* = 0.017		1.01 (0.97–1.05),*p* = 0.5883		1.07 (1.0-1.14),*p* = 0.0352	1.05 (1.01–1.10),*p* = 0.0165
Sex	Female		0.68 (0.39–1.2),*p* = 0.1871	0.39 (0.16-1.0),*p* = 0.0493		1.17 (0.54–2.52),*p* = 0.6890		0.85 (0.23–3.06),*p* = 0.7983	1.06 (0.54–2.54),*p* = 0.8915
	Male		ref	ref		ref		ref	ref
HL subtype	NLPHL		0.64 (0.26–1.54),*p* = 0.3170	0.98 (0.33–2.95),*p* = 0.9743		0.31 (0.04–2.39),*p* = 0.2628		N/A	N/A
	cHL		ref	ref		ref		ref	ref
CCI			0.62 (0.29–1.33),*p* = 0.2176	1.37 (1.11–1.68),*p* = 0.0028		0.92 (0.53–1.58),*p* = 0.7624		1.40 (1.05–1.87),*p* = 0.0209	1.45 (1.19–1.77),*p* < 0.001
Diagnosis year			0.96 (0.89–1.03),*p* = 0.2306	0.95 (0.85–1.05),*p* = 0.3051		1.01 (0.93-1.0),*p* = 0.8018		1.04 (0.85–1.28),*p* = 0.6862	0.99 (0.88–1.12),*p* = 0.9032
Radiation status	Yes		0.52 (0.22–1.25),*p* = 0.1429	4.05 (1.62–10.14),*p* = 0.0028		0.76 (0.27–2.13),*p* = 0.6070		1.86 (0.42–8.28),*p* = 0.4165	1.61 (0.52–4.95),*p* = 0.4089
	No		ref	ref		ref		ref	ref
Stem cell transplant	Yes		2.29 (0.80–6.59),*p* = 0.1242	N/A		1.67 (0.38–7.27),*p* = 0.4964		N/A	1.60 (0.53–4.82),*p* = 0.4056
	No		ref	ref		ref		ref	ref
B) in the older (≥ 50 years) group									
Age at diagnosis			1.07 (1.05–1.1),*p* < 0.001	1.10 (1.07–1.12),*p* < 0.001		1.07 (1.03–1.10),*p* < 0.001		1.03 (0.98–1.08),*p* = 0.2083	1.11 (1.08–1.13),*p* < 0.001
Sex	Female		0.44 (0.30–0.67),*p* < 0.001	0.71(0.47–1.06),*p* = 0.0948		0.53 (0.32-1.0),*p* = 0.0956		0.29 (0.09–0.87),*p* = 0.0277	0.69 (0.47–1.01),*p* = 0.0541
	Male		ref	ref		ref		ref	ref
HL subtype	NLPHL		1.22 (0.78–1.89),*p* = 0.3852	1.02 (0.62–1.66),*p* = 0.9473		0.52 (0.22–1.24),*p* = 0.1408		0.44 (0.13–1.52),*p* = 0.1950	0.54 (0.30–0.94),*p* = 0.0301
	cHL		ref	ref		ref		ref	ref
CCI			1.08 (0.94–1.23),*p* = 0.2756	1.02 (0.89–1.18),*p* = 0.7498		1.27 (1.09–1.49),*p* = 0.0026		1.02 (0.73–1.43),*p* = 0.9009	1.11 (0.99–1.24),*p* = 0.084
Diagnosis year			0.97 (0.93-1.0),*p* = 0.0773	1.00 (0.96–1.04),*p* = 0.9493		1.07 (1.0-1.15),*p* = 0.0551		1.02 (0.92–1.12),*p* = 0.7179	1.0 (0.96–1.04),*p* = 0.9773
Radiation status		Yes	0.75 (0.45–1.27),*p* = 0.2858	0.77 (0.46–1.31),*p* = 0.3396		1.35 (0.69–2.63),*p* = 0.3789		0.52 (0.15–1.82),*p* = 0.3063	0.72 (0.43–1.19),*p* = 0.1989
		No	ref	ref		ref		ref	ref
Stem cell transplant		Yes	2.17 (0.77–6.11),*p* = 0.1419	1.93 (0.59–6.36),*p* = 0.2789		1.89 (0.43–8.28),*p* = 0.4006		N/A	N/A
		No	ref	ref		ref		ref	ref

*HR* hazards ratio, *CI* confidence interval, *HL* Hodgkin lymphoma, *NLPHL* nodular lymphocyte-predominant Hodgkin lymphoma, *cHL* classical Hodgkin lymphoma, *CCI*  Charlson comorbidity index, N/A  not available

### Pulmonary diseases

#### Pneumonia

**Fig. 1:**
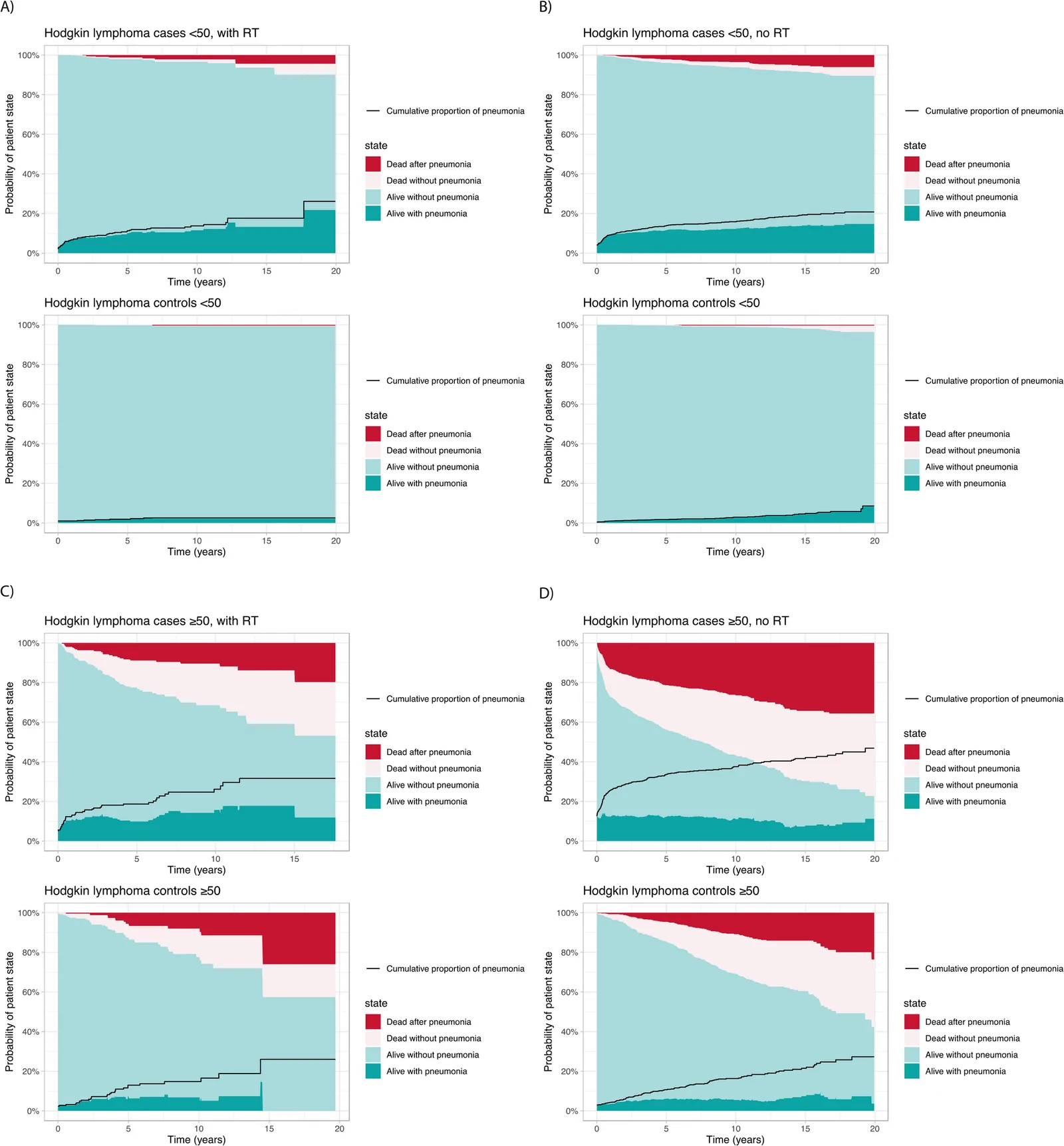
Aalen-Johanssen multi-state model for the occurrence of pneumonia (Table S3) in (**a**) patients < 50 years of age with radiotherapy (**b**) patients < 50 years without radiotherapy (**c**) patients ≥ 50 years of age with radiotherapy (**d**) patients ≥ 50 years without radiotherapy

In the younger age group, the incidence rate of pneumonia was substantially higher in patients than in controls both at baseline (RR 5.91 (3.11–11.32), *p* < 0.001) and for years thereafter (RR 4.89 (3.58–6.71), *p* < 0.001) (Table 4A and Fig. 1AB). Older age at diagnosis (HR 1.02, 95% CI 1.00-1.03, *p* = 0.0142), higher CCI at baseline (HR 1.21, 95% CI 1.09–1.34, *p* < 0.001), and stem cell transplant (SCT) (HR 5.22, 95% CI 3.38-8.0, *p* < 0.001) were linked with a higher risk of pneumonia (Table 5A). In the younger age group, the proportional hazard assumption was not fulfilled for RT status, but the results still give a valid representation of average risks over time. In the older age group, the incidence rate of pneumonia was also notably high among patients compared to controls at baseline (RR 3.87 (2.61–5.71), *p* < 0.001) and during follow-up (RR 2.51 (2.02–3.11), *p* < 0.001) (Table 4B and Fig. 1CD) and the risk factors were similar to those in the younger group, except for CCI. Additionally, patients with the NLPHL subtype (HR 0.49, 95% CI 0.33–0.72, *p* < 0.001) and irradiated patients (HR 0.60, 95% CI 0.40–0.89, *p* = 0.011) had a lower risk for pneumonia (Table 5B, Table S3).

### Other lung infections

The incidence rate of other lung infections in the younger age group was notably higher in the HL group than in the control group (RR 2.99 (1.89–4.67), *p* < 0.001) (Table 4A). Especially SCT was associated with almost nine-fold higher risk (HR 8.73, 95% CI 4.49–16.69, *p* < 0.001) for other lung infections (Table 5A). In addition, the occurrence of other lung infections was higher in older patients than in controls at baseline (RR 3.00 (1.45–6.2), *p* = 0.003) and during follow-up (RR 3.51 (2.16–5.75), *p* < 0.001) (Table 4B). The NLPHL subtype was linked with a lower risk (HR 0.37, 95% CI 0.16–0.87, *p* < 0.023) for other lung infections (Table 5B).

### Pulmonary embolism

In younger patients, the incidence rate of pulmonary embolism was 7.78 times higher than that in controls (*p* < 0.001) (Table 4A). Additionally, older age at diagnosis was associated with a slightly increased risk (HR 1.05, 95% CI 1.01–1.09, *p* = 0.0077) (Table 5A). Also, both at baseline (RR 6.0 (1.35–26.57), *p* = 0.019) and during follow-up (RR 2.00 (1.17–3.38), *p* = 0.009), the incidence rate of pulmonary embolism was higher in older patients than in controls (Table 4B). A later year of diagnosis was associated with an increased risk (HR 1.11, 95% CI 1.03–1.19, *p* = 0.0059) for pulmonary embolism in the older patient group (Table 5B).

### Other lung diseases

Among patients under 50 years of age, a significantly higher incidence rate of other lung diseases was observed compared to controls (RR 2.27 (1.6–3.23), *p* < 0.001) (Table 4A). Older age at diagnosis (HR 1.03, 95% CI 1.01–1.05, *p* = 0.0102) and especially female sex (HR 1.69, 95% CI 1.11–2.58, *p* = 0.0149) were associated with an elevated risk of other lung diseases (Table 5A). In the older group, the incidence of other lung diseases was also significantly higher in patients than in controls (RR 1.83 (1.26–2.64), *p* = 0.002) (Table 4B). Additionally, a later calendar year of diagnosis was associated with an increased risk of developing these conditions (HR 1.07, 95% CI 1.01–1.14, *p* = 0.0179) (Table 5B).

**Table 4:** Cumulative incidence (%) of newly diagnosed pulmonary diseases and corresponding rate ratios (RR) of incidence rates in the patients with HL

	Group	Follow-up time (years)						BaselineRR,p-value	Follow-up RR,p-value
		0	1	5	10	15	20		
A)n the younger (< 50 years) group									
Cum. incidence, (%)Pneumonia	HL	0	5.0	9.6	11.7	15.3	17.2	5.91 (3.11–11.32),*p* < 0.001	4.89 (3.58–6.71),*p* < 0.001
	control	0	0.3	1.2	2.2	3.9	7.4		
Cum. incidence, (%)Other lung infections*	HL	0	1.6	3.0	3.7	5.5	5.8	1.57 (0.61–4.02),*p* = 0.35	2.99 (1.89–4.67),*p* < 0.001
	control	0	0.2	0.8	0.9	2.4	3.1		
Cum. incidence, (%)Pulmonary embolism	HL	0	0.7	1.3	1.5	1.8	2.6	†	7.78 (2.75–21.95),*p* < 0.001
	control	0	0	0	0.2	0.2	0.5		
Cum. incidence, (%)Other lung diseases**	HL	0	1.2	3.6	5.0	7.3	8.9	1.38 (0.86–2.20),*p* = 0.187	2.27 (1.60–3.23),*p* < 0.001
	control	0	0.5	1.3	2.4	3.3	4.7		
B) in the older (≥ 50 years) group									
Cum. incidence, (%)Pneumonia	HL	0	11.9	19.3	24.0	29.3	34.7	3.87 (2.61–5.71),*p* < 0.001	2.51 (2.02–3.11),*p* < 0.001
	control	0	1.2	8.3	13.3	19.0	24.5		
Cum. incidence, (%)Other lung infections*	HL	0	2.6	5.0	5.6	7.9	8.5	3.00 (1.45–6.20),*p* = 0.003	3.51 (2.16–5.75),*p* < 0.001
	control	0	0.4	1.4	2.5	4.2	4.2		
Cum. incidence, (%)Pulmonary embolism	HL	0	2.0	3.2	3.7	3.9	3.9	6.0 (1.35–26.57),*p* = 0.019	2.00 (1.17–3.38),*p* = 0.009
	control	0	0.2	1.7	2.4	3.7	4.3		
Cum. incidence, (%)Other lung diseases**	HL	0	2.0	4.2	5.9	9.4	10.8	1.57 (1.00-2.46),*p* = 0.055	1.83 (1.26–2.64),*p* = 0.002
	control	0	0.5	3.1	5.8	6.9	6.9		

RR  rate ratio† not available due to zero events in cases and/or controls

**Table 5:** Cox multivariable model fit for hazard for newly diagnosed pulmonary diseases in patients with HL in

Covariate		HR, (95% CI), p-value				
		Pneumonia	Other lung infections	Lung cancer	Pulmonary embolism	Other lung diseases
A) in the younger (< 50 years) group						
Age at diagnosis		1.02 (1.0-1.03), *p* = 0.0142	1.01 (0.98–1.03), *p* = 0.5734	1.02 (0.94–1.11), *p* = 0.5588	1.05 (1.01–1.09), *p* = 0.0077	1.03 (1.01–1.05), *p* = 0.0102
Sex	*Female*	0.99 (0.76–1.31), *p* = 0.9637	1.69 (1.04–2.74), *p* = 0.0347	0.27 (0.03–2.40), *p* = 0.2399	1.04 (0.49–2.18), *p* = 0.9275	1.69 (1.11–2.58), *p* = 0.0149
	*Male*	ref	ref	ref	ref	ref
HL subtype	*NLPHL*	0.90 (0.58–1.41), *p* = 0.6529	1.25 (0.57–2.71), *p* = 0.5772	1.20 (0.12–11.53), *p* = 0.8771	0.90 (0.30–2.71), *p* = 0.8508	0.78 (0.37–1.67), *p* = 0.5281
	*cHL*	ref	ref	ref	ref	ref
CCI		1.21 (1.09–1.34), *p* < 0.001	1.07 (0.84–1.37), *p* = 0.5787	N/A	1.00 (0.69–1.47), *p* = 0.9803	0.94 (0.70–1.26), *p* = 0.6730
Diagnosis year		1.02 (1.0-1.05), *p* = 0.1057	1.04 (0.99–1.09), *p* = 0.1463	0.87 (0.65–1.17), *p* = 0.3510	1.00 (0.93–1.08), *p* = 0.9660	1.00 (0.95–1.05), *p* = 0.9825
Radiation status	*Yes*	0.69 (0.46–1.01), *p* = 0.0573	0.84 (0.44–1.61), *p* = 0.5990	N/A	0.73 (0.24–2.22), *p* = 0.5772	0.82 (0.47–1.44), *p* = 0.4985
	*No*	ref	ref	ref	ref	ref
Stem cell transplant	*Yes*	5.22 (3.38–8.09), *p* < 0.001	8.73 (4.49–16.97), *p* < 0.001	N/A	1.31 (0.17–10.05), *p* = 0.7978	1.09 (0.40–3.03), *p* = 0.8616
	*No*	ref	ref	ref	ref	ref
B) in the older (≥ 50 years) group						
Age at diagnosis		1.04 (1.03–1.06), *p* < 0.001	1.02 (0.99–1.05), *p* = 0.1299	0.99 (0.92–1.05), *p* = 0.5885	1.00 (0.96–1.03), *p* = 0.8648	1.01 (0.98–1.04), *p* = 0.5812
Sex	*Female*	0.87 (0.66–1.16), *p* = 0.1664	0.68 (0.38–1.19), *p* = 0.1735	0.22 (0.05–1.01), *p* = 0.0509	0.87 (0.43–1.78), *p* = 0.7058	1.29 (0.77–2.15), *p* = 0.3357
	*Male*	ref	ref	ref	ref	ref
HL subtype	*NLPHL*	0.49 (0.33–0.72), *p* < 0.001	0.37 (0.16–0.87), *p* = 0.0231	0.55 (0.15–1.98), *p* = 0.3630	0.41 (0.14–1.16), *p* = 0.0922	0.79 (0.42–1.50), *p* = 0.4752
	*cHL*	ref	ref	ref	ref	ref
CCI		1.07 (0.97–1.16), *p* = 0.1664	1.04 (0.87–1.26), *p* = 0.6573	0.65 (0.29–1.46), *p* = 0.2929	1.16 (0.95–1.42), *p* = 0.1429	1.09 (0.92–1.29), *p* = 0.3263
Diagnosis year		1.01 (0.98–1.04), *p* = 0.4380	1.03 (0.97–1.08), *p* = 0.3740	0.99 (0.89–1.10), *p* = 0.8683	1.11 (1.03–1.19), *p* = 0.0059	1.07 (1.01–1.14), *p* = 0.0179
Radiation status	*Yes*	0.60 (0.40–0.89), *p* = 0.011	0.62 (0.30–1.29), *p* = 0.2003	0.58 (0.12–2.74), *p* = 0.49	0.49 (0.19–1.29), *p* = 0.1487	0.52 (0.25–1.07), *p* = 0.0756
	*No*	ref	ref	ref	ref	ref
Stem cell transplant	*Yes*	2.38 (1.14–4.95), *p* = 0.0204	0.77 (0.1–5.72), *p* = 0.7988	1.93 (0.24–15.83), *p* = 0.5405	N/A	0.54 (0.07-4.00), *p* = 0.5458
	*No*	ref	ref	ref	ref	ref

*HR* hazards ratio, *CI*  confidence interval, *HL*  Hodgkin lymphoma, *NLPHL*  nodular lymphocyte-predominant Hodgkin lymphoma, *cHL*  classical Hodgkin lymphoma, *CCI*  Charlson comorbidity index, RR rate ratio

## Discussion

This was a large real-world study describing long-term adverse events after modern HL treatment. We observed a 3.8-fold higher incidence rate of CADs in patients aged < 50 years than in controls. Moreover, patients aged ≥ 50 years at diagnosis had a 1.8-fold higher occurrence of valvular heart disease and a 1.68-fold higher risk of chronic heart failure than their controls. The most notable findings were an almost 6-fold higher incidence rate of pneumonia at baseline and a 5-fold higher occurrence of pneumonia in younger patients than in controls during long-term follow-up. Remarkably, according to the current treatment guidelines, late cardiopulmonary morbidity in long-term survivors of HL remains a great challenge.

After the introduction of multi-agent chemotherapy and extended-field radiation in the 1970s [22, 27], HL became one of the first curable malignancies, but the cost of severe long-term sequelae resulted in excess late mortality [28]. Subsequently, less toxic ABVD regimen was introduced, and the number of administered chemotherapy cycles were shortened, especially in limited-stage diseases [29, 30], and treatment for the advanced disease has been guided by PET-CT [31].

Also, mantle field radiotherapy has been a major source of toxicity linked to an increased risk of long-term cardiovascular toxicities and a 3.1-fold risk of death caused by heart disease [32]. Fortunately, smaller radiation fields and lower doses of radiotherapy have been shown to offer similar efficacy in limited-stage diseases [33, 34] and modern imaging and radiotherapy techniques increasingly spare the normal tissue [35]. Due to the nature of our study, we did not have the doses or sites for radiotherapy available, but international guidelines in the treatment have been followed [3]. Volumetric-modulated arc therapy has been reported to reduce the mean lung and heart doses compared to 3D-conformal radiotherapy [36], thus limiting a risk of cardiovascular disease [14] and incorporating deep inspiration breath-hold appears to be beneficial [37].

Despite major advancements in HL treatment protocols, the 10-year cumulative incidence of CAD was still significantly elevated among young HL survivors compared to controls (3.0 vs. 0.8%), but less than that reported in those treated before the 2000s [15, 38]. In particular, the rate ratios for CAD or infarction (RR 3.8) and CHF (RR 2.72) remained higher than those in controls but statistically significant association between the diagnosis period and cardiopulmonary complications were not found. Older age and higher CCI seemed to be linked with a remarkably increased risk of arrhythmia, cardiomyopathy, and CHF. In addition, we detected a slightly lower incidence of 3.6% of CVDs in non-irradiated younger patients compared with the 4.5% reported by Galper et al. after radiotherapy [39]. In our study, radiotherapy was associated only with a 4-fold risk of arrhythmias in younger patients. Moreover, a possible imbalance between the groups regarding the chemotherapy regimen used, possible variable sites and doses of radiotherapy, and limited follow-up time as well as small patient numbers and underreporting of RT in the earlier years may have hampered this result. In addition, excluding patients with pre-existing diagnosis of comorbidities bias the analysis, but the effect of this is expected to be minimal, as the patients with HL continued to have higher rates of comorbidities during follow up than controls, highlighting the difference between cases and controls. Among older patients, the association between late morbidity and HL was less clear, partly because of the healthy survivor effect that affects the results in addition to more intensive follow-up of HL patients after treatment [40].

Valvular heart diseases showed a notable decrease in incidence compared to patients treated before the 2000s (1.5% in the younger group and 5.2% in the older group vs. previously published 8%) [41]. Anthracycline-based chemotherapy has proven to increase the risk of valvular heart disease [15], likewise the mediastinal RT [15, 42]. This contrasts with our observations, where the 1.7-fold higher incidence rate for valvular problems was limited to non-irradiated patients, possibly due to a lower peak dose of radiation in the valvular region using arc techniques [35].

Although the incidence of heart disease among younger patients was much lower than the 37% reported in a previous study [14], it was still higher than that in controls. This highlights the importance of preventive measures, early diagnosis, and patient-specific treatment decisions for HL. These include screening for early decrease in ejection fraction with echocardiography during the first year after the start of chemotherapy, using biomarkers, and using prophylactic medication in high-risk HL patients [43]. Cardiovascular risk factors should be treated also in long-term asymptomatic survivors [43]. A radiation-free regimen may be considered in patients with large mediastinal masses [44]. In addition, efforts should be made to optimise the dose distribution, use respiratory gating in radiotherapy [45], and define the possibilities of proton therapy. Dexrazoxane or liposomal anthracyclines may be considered instead of doxorubicin in patients with an elevated risk of CVD [43]. In the future, anthracycline-free regimens such as brentuximab vedotin and immuno-oncology agents may play a role in earlier treatment lines.

An interesting result of our study was the up to fivefold increase in the incidence rate of pneumonia and over threefold increase in the occurrence of other lung infections during long-term follow-up in patients compared to controls. However, the cumulative incidence of pulmonary infections such as pneumonia after a 10-year follow-up of 11.7% in the younger group and 24.0% in the older group observed in our study was remarkably lower than the 57% reported before the 2000s [28]. The 5.9-fold incidence rate of pneumonia at baseline is a possible predictor of malignancy [21]. Patients with NLPHL had a lower risk of pneumonia, likely due to lighter treatments. A higher risk of pulmonary infections is most likely caused by immunosuppression and neutropenia after high-dose therapy, whereas principally weakened immune system due to the disease itself and therapy in the non-transplanted patients are predisposed to infections.

A recent Danish study showed that patients with classical HL treated with current treatment modalities are more likely to develop interstitial and obstructive lung diseases than the background population [46]. In the present study, only a limited number got those diagnoses during modern treatment modalities. In addition, bleomycin use has been associated with the risk of impaired respiratory function for decades [47]. These comorbidities may also predispose patients to pulmonary infections due to impaired host immune response and colonisation by micro-organisms [48], which may be partly responsible for the elevated risk of infections that persist for years. The recommended vaccines (pneumococcal, influenza) may reduce the risk of infection [9]. The omission of bleomycin after two chemotherapy courses from the treatment regimens did not negatively affect the overall survival of patients with positron emission tomography (PET)-negative disease [17, 45, 49], and a critical evaluation of the role of bleomycin in the treatment protocols is warranted. Conclusions on the necessity of bleomycin use need a prospective study.

This study has some limitations. There is a possibility of missing or inaccurate data owing to inconsistencies in recording practices. Overlap in the records was also possible, although its impact on the results was minimal. Additionally, due to the lack of data on disease stageing, possible bulky disease, hospital-administrated medications, the therapy profiles of the study cohort and the possible effects of changes in treatment practices could not be analyzed within this study. In addition, the potential underreporting of the administered radiotherapy treatments may limit the accuracy of the results. Moreover, no data were available regarding CAD risk factors like smoking habits or asymptomatic and undiagnosed long-term consequences in patients or controls. Also, comorbidities at diagnosis may be more intensively registered in patients than controls which may have impacted CCI scores and comorbidities observed around the HL diagnosis. Finally, although our data coverage was 20 years, the average follow-up time was less than 10 years, which may have underestimated late toxicities, and the results should be interpreted with this in mind. However, the strengths of this study include real-life data from current treatment practices involving a large population, reliable registries [50, 51] as the source material, and clearly matched control groups. In addition, treatment practices comparable to national and international guidelines have been widely used in Finland [3, 52].

In conclusion, remarkable late toxicity remains in long-term HL survivors. The high incidence of pulmonary infections in both age groups, CAD, and valvular heart disease, especially in older patients with HL, remains a challenge. The importance of long-term surveillance, preventive strategies, and early detection of late toxicity in survivors of HL cannot be overstated. Therefore, reconsidering the optimal treatment modalities for HL is warranted, especially in older patients.

## Supplementary Information

Below is the link to the electronic supplementary material.

Supplementary Material 1

## Data Availability

This study is based on the secondary use of healthcare register data. Data can be acquired with permission by following the guidelines and application processes of the registries.
