# Supplementary material for: Late cardiopulmonary morbidity in patients with Hodgkin lymphoma: a retrospective nationwide registry study
Source: Ann Hematol. 2026 Jul 22;105(11):462. doi: 10.1007/s00277-026-07193-9 (PMC13677623; doi:10.1007/s00277-026-07193-9)
Supplement: Supplementary file 1 [file 277_2026_7193_MOESM1_ESM.docx]

Supplementary files

Late cardiopulmonary morbidity in patients with Hodgkin lymphoma: a retrospective nationwide registry study

Noora Hannuksela^1^, Outi Kuittinen^1,2^, Aino Rönkä^1,2^, Anna Anttalainen^3^, Liisa Ukkola-Vuoti^3^, Iiro Toppila^3^, Johanna Vikkula^3^, Jan Seppälä^4^, Kristiina Vuolukka^2^, Katja Marin^2^, Hanne Kuitunen^5^, Tatu Miettinen^6^, Anu Partanen^7^

1 Oncology, Faculty of Health Medicine, School of Medicine, Institute of Clinical Medicine, University of Eastern Finland, Kuopio, Finland

2 Department of Oncology, Kuopio University Hospital, Kuopio, Finland

3 Medaffcon Oy, Espoo, Finland

4 Department of Oncology, Department of Radiotherapy, Kuopio University Hospital, Kuopio, Finland

5 Cancer Centre, Oulu University Hospital, Oulu, Finland

6 Takeda Oy, Helsinki, Finland

7 Department of Medicine, Kuopio University Hospital, Kuopio, Finland

Correspondence

Noora Hannuksela, Oncology, Faculty of Health Medicine, School of Medicine, Institute of Clinical Medicine, University of Eastern Finland, Kuopio, Finland

Email: nhannuks@student.uef.fi

Figure S1. Flowchart of data collection

**CONTROL POPULATION IDENTIFICATION**

All individuals without any lymphoma diagnosis before 2020 nationwide at the Digital and Population Data Services Agency

**CONTROL GROUP FORMATION:**

Ratio 1:1

Matched by age, sex, and region in Finland

Alive at case index (the time of diagnosis)

**PATIENT IDENTIFICATION**

Patients with HL diagnosis (ICD-10:C81*) between Jan 1^st^ 2000 and Dec 31^st^ 2019 nationwide in Finland. Collected from the Finnish Cancer Registry.

n = 2912

**DATA ENRICHMENT:**

The personal identification number which is unique for each Finnish resident was utilized for data linkage between the six registries.

**Older group**	≥50 years old patients at the time of diagnosis	n = 1057

**Younger group**

<50 years old patients at the time of diagnosis

n = 1855

**CONTROL GROUPS**

HL control groups for <50 years old radiated and radiation-free patients at the time of diagnosis

HL control groups for ≥50 years old radiated anad radiation-free patients at the time of diagnosis

**Radiation-free**

n = 804

**Radiated**

n = 253

**Radiation-free**

n = 1316

**Radiated**

n = 539

**Younger (<50) group**

**Older (>50) group**

**ANALYSIS GROUPS**

**Controls for the radiated**

n = 539

**Controls for the radiation-free**

n = 1316

**Controls for the radiated**

n = 253

**Controls for the radiation-free**

n = 804

Figure S2. Aalen-Johanssen multi-state model for the occurrence of newly diagnosed coronary artery disease or infarction in a) patients <50 years of age with radiotherapy b) patients <50 years without radiotherapy c) patients >50 years of age with radiotherapy d) patients >50 years without radiotherapy

A)

B)

C)

D)

Figure S3. Aalen-Johanssen multi-state model for the occurrence of valvular heart disease (Table S2) in a) patients <50 years of age with radiotherapy b) patients < 50 years without radiotherapy c) patients ≥50 years of age with radiotherapy d) patients ≥50 years without radiotherapy.

Table S1. ICD-10 codes for comorbidities

| **Comorbidities** | **ICD-10 diagnosis codes** |
| --- | --- |
| **Coronary artery disease or infarct** | I25 chronic ischemic heart disease, I20 angina pectoris, I21 acute myocardial infarction |
| **Atrial arrhythmia** | I48 atrial fibrillation and flutter |
| **Valvular heart disease** | I35 non-rheumatic aortic valve disease, I34 non-rheumatic mitral valve disease, I36 non-rheumatic tricuspidal valve disease |
| **Cardiomyopathy** | I42 Cardiomyopathy |
| **Congestive heart failure** | I50 Heart failure, J81 Pulmonary edema |
| **Pneumonia** | J18 pneumonia, organism unspecified, J15 bacterial pneumonia, not elsewhere classified, J12 viral pneumonia, not elsewhere classified, B59 pneumocystosis, J13 pneumonia due to Streptococcus pneumoniae, J17 pneumonia in diseases classified elsewhere |
| **Other lung infections** | J21 acute bronchiolitis, J20 & J40 bronchitis, A15 & A16 & A19 tuberculosis |
| **Pulmonary embolism** | I26 pulmonary embolism |
| **Other lung diseases** | J69 pneumonitis, J42 chronic bronchitis, J67 hypersensitivity pneumonitis, J45 asthma, J48 other interstitial pulmonary diseases, J43 emphysema, J47 bronchiectasis |

Table S2. Cumulative incidence (%) of newly diagnosed CVDs and corresponding rate ratios (RR) of incidence rates in HL patients by radiation status, during the follow-up, with the baseline diagnoses excluded

A) in the younger (<50 years) group

|  | **Radiation status** | **Group** | **Follow-up time (years)** | | | | | | **Baseline**  **RR,**  **p-value** | **Follow-up RR p-value** |
| --- | --- | --- | --- | --- | --- | --- | --- | --- | --- | --- |
|  |  |  | **0** | **1** | **5** | **10** | **15** | **20** |  |  |
| **Cum. incidence,** (%) coronary artery disease or infract | *Radiated* | *HL* | 0.0 | 0.0 | 0.4 | 0.9 | 0.9 | 19.3 | † | 2.05 (0.51-8.26),  p=0.309 |
|  |  | *control* | 0.0 | 0.0 | 0.3 | 0.6 | 0.6 | 9.7 |  |  |
|  | *Radiation-free* | *HL* | 0.0 | 0.4 | 1.5 | 3.6 | 5.7 | 6.7 | 4 (0.45-36.06),  p=0.215 | 4.25 (2.28-7.98),  p<0.001 |
|  |  | *control* | 0.0 | 0.1 | 0.4 | 0.9 | 1.3 | 1.8 |  |  |
| **Cum. incidence,** (%) arrhythmia | *Radiated* | *HL* | 0.0 | 0.6 | 1.1 | 2.8 | 6.1 | 6.1 | † | 6.16 (1.39-27.37),  p=0.017 |
|  |  | *control* | 0.0 | 0.0 | 0.0 | 0.8 | 0.8 | 0.8 |  |  |
|  | *Radiation-free* | *HL* | 0.0 | 0.2 | 0.4 | 0.5 | 1.3 | 2.9 | 0.67 (0.11-3.95),  p=0.657 | 1.46 (0.65-3.27),  p=0.364 |
|  |  | *control* | 0.0 | 0.0 | 0.5 | 0.8 | 0.9 | 1.4 |  |  |
| **Cum. incidence,** (%) valvular heart disease | *Radiated* | *HL* | 0.0 | 0.6 | 1.0 | 1.4 | 1.4 | 1.4 | 0.5 (0.05-5.48),  p=0.571 | 6.02(0.72-49.74),  p=0.097 |
|  |  | *control* | 0.0 | 0.0 | 0.0 | 0.0 | 1.9 | 1.9 |  |  |
|  | *Radiation-free* | *HL* | 0.0 | 0.2 | 0.8 | 1.5 | 2.2 | 3.0 | † | † |
|  |  | *control* | 0.0 | 0.0 | 0.2 | 0.2 | 0.4 | 0.6 |  |  |
| **Cum. incidence,** (%) cardiomyopathy | *Radiated* | *HL* | 0.0 | 0.0 | 0.0 | 0.4 | 1.8 | 1.8 | † | 3.08 (0.32-29.49),  p=0.33 |
|  |  | *control* | 0.0 | 0.0 | 0.0 | 0.5 | 0.5 | 0.5 |  |  |
|  | *Radiation-free* | *HL* | 0.0 | 0.0 | 0.2 | 0.4 | 0.6 | 1.2 | † | 1.82 (0.53-6.26),  p=0.339 |
|  |  | *control* | 0.0 | 0.0 | 0.0 | 0.3 | 0.3 | 0.6 |  |  |
| **Cum. incidence,** (%) congestive heart failure | *Radiated* | *HL* | 0.0 | 0.2 | 0.4 | 0.4 | 3.6 | 11.6 | † | 6.16 (0.74-51.25),  p=0.092 |
|  |  | *control* | 0.0 | 0.0 | 0.0 | 0.4 | 0.4 | 0.4 |  |  |
|  | *Radiation-free* | *HL* | 0.0 | 0.1 | 0.5 | 0.9 | 1.5 | 2.1 | † | 2.23 (0.90-5.48),  p=0.08 |
|  |  | *control* | 0.0 | 0.0 | 0.0 | 0.4 | 1.0 | 1.0 |  |  |
| † not available due to zero events in cases and/or controls  RR = rate ratio | | | | | | | | | | |

B) in the older (≥50 years) group

|  | **Radiation status** | **Group** | **Follow-up time (years)** | | | | | | **Baseline**  **RR,**  **p-value** | **Follow-up RR,**  **p-value** |
| --- | --- | --- | --- | --- | --- | --- | --- | --- | --- | --- |
|  |  |  | **0** | **1** | **5** | **10** | **15** | **20** |  |  |
| **Cum. incidence,** (%) Coronary artery disease or infarct | *Radiated* | *HL* | 0.0 | 1.2 | 5.4 | 11.2 | 21.3 | 21.3 | 1.21 (0.60-2.45),  p=0.591 | 1.50 (0.77-2.90),  p=0.210 |
|  |  | *control* | 0.0 | 1.2 | 4.9 | 8.7 | 10.9 | 10.9 |  |  |
|  | *Radiation-free* | *HL* | 0.0 | 4.9 | 9.5 | 13.3 | 16.8 | 19.9 | 0.61 (1.14-2.30),  p=0.009 | 1.27 (0.97-1.67),  p=0.081 |
|  |  | *control* | 0.0 | 3.2 | 10.3 | 16.4 | 20.9 | 23.8 |  |  |
| **Cum. incidence,** (%) Arrhythmia | *Radiated* | *HL* | 0.0 | 3.2 | 7.5 | 12.4 | 16.3 | 16.3 | 1.00 (0.45-2.23),  p=1.000 | 1.31 (0.71-2.41),  p=0.385 |
|  |  | *control* | 0.0 | 0.4 | 7.1 | 9.9 | 14.9 | 27.1 |  |  |
|  | *Radiation-free* | *HL* | 0.0 | 2.8 | 7.9 | 11.4 | 13.6 | 14.9 | 1.19 (0.83-1.69),  p=0.333 | 1.10 (0.82-1.47),  p=0.560 |
|  |  | *control* | 0.0 | 1.6 | 8.7 | 14.7 | 20.6 | 26.3 |  |  |
| **Cum. incidence,** (%) Valvular heart disease | *Radiated* | *HL* | 0.0 | 1.6 | 4.1 | 8.8 | 18.2 | 33.1 | 0.20 (0.02-1.73),  p=0.142 | 1.90 (0.83-4.32),  p=0.120 |
|  |  | *control* | 0.0 | 0.4 | 2.5 | 5.3 | 7.5 | 7.5 |  |  |
|  | *Radiation-free* | *HL* | 0.0 | 1.2 | 3.1 | 4.3 | 5.4 | 8.0 | 1.19 (0.61-2.31),  p=0.613 | 1.73 (1.06-2.83),  p=0.031 |
|  |  | *control* | 0.0 | 0.6 | 1.0 | 1.4 | 1.4 | 1.4 |  |  |
| **Cum. incidence,** (%) Cardiomyopathy | *Radiated* | *HL* | 0.0 | 0.4 | 0.8 | 2.9 | 2.9 | 2.9 | 2.00 (0.36-10.97),  p=0.423 | 4.28 (0.47-38.29),  p=0.193 |
|  |  | *control* | 0.0 | 0.0 | 0.5 | 0.5 | 0.5 | 0.5 |  |  |
|  | *Radiation-free* | *HL* | 0.0 | 0.0 | 0.0 | 0.5 | 0.5 | 0.5 | 1.00 (0.35-2.83),  p=1.000 | 3.31 (1.46-7.56),  p=0.005 |
|  |  | *control* | 0.0 | 0.1 | 0.6 | 1.3 | 1.3 | 2.5 |  |  |
| **Cum. incidence,** (%) Congestive heart failure | *Radiated* | *HL* | 0.0 | 3.6 | 7.5 | 14.4 | 16.0 | 35.8 | 2.00 (0.50-8.02),  p=0.327 | 1.90 (0.99-3.62),  p=0.054 |
|  |  | *control* | 0.0 | 0.8 | 5.2 | 9.1 | 17.0 | 32.7 |  |  |
|  | *Radiation-free* | *HL* | 0.0 | 2.7 | 8.2 | 12.5 | 15.8 | 18.9 | 2.05 (1.21-3.49),  p=0.008 | 1.54 (1.15-2.06),  p=0.004 |
|  |  | *control* | 0.0 | 0.2 | 0.4 | 0.4 | 3.6 | 11.6 |  |  |
| RR = rate ratio | | | | | | | | | | |

Table S3. Cumulative incidence (%) of newly diagnosed pulmonary diseases and corresponding rate ratios (RR) of incidence rates in HL patients by radiation status, during the follow-up, with the baseline diagnoses excluded

A) in the younger (<50 years) group

|  | **Radiation status** | **Group** | **Follow-up time (years)** | | | | | | **Baseline**  **RR,**  **p-value** | **Follow-up RR,**  **p-value** |
| --- | --- | --- | --- | --- | --- | --- | --- | --- | --- | --- |
|  |  |  | **0** | **1** | **5** | **10** | **15** | **20** |  |  |
| **Cum. incidence,** (%)  Pneumonia | *Radiated* | *HL* | 0.0 | 4.5 | 9.1 | 11.5 | 15.4 | 23.9 | 2.40 (0.85-6.81),  p=0.100 | 9.42 (4.04-21.82),  p<0.001 |
|  |  | *control* | 0.0 | 0.0 | 0.9 | 1.5 | 1.5 | 1.5 |  |  |
|  | *Radiation-free* | *HL* | 0.0 | 5.2 | 9.8 | 11.7 | 15.3 | 16.8 | 8.83 (3.81-20.55),  p<0.001 | 4.21 (2.97-6.01),  p<0.001 |
|  |  | *control* | 0.0 | 0.5 | 1.3 | 2.4 | 4.3 | 8.1 |  |  |
| **Cum. incidence,** (%)  Other lung infections* | *Radiated* | *HL* | 0.0 | 2.1 | 3.7 | 4.5 | 7.6 | 7.6 | 2.00 (0.36-10.97),  p=0.423 | 3.59 (1.46-8.86),  p=0.006 |
|  |  | *control* | 0.0 | 0.4 | 1.3 | 1.3 | 1.3 | 1.3 |  |  |
|  | *Radiation-free* | *HL* | 0.0 | 1.40 | 2.8 | 3.5 | 5.2 | 5.5 | 1.40 (0.44-4.47),  p=0.565 | 2.79 (1.65-4.76),  p<0.001 |
|  |  | *control* | 0.0 | 0.2 | 0.6 | 0.7 | 2.4 | 3.2 |  |  |
| **Cum. incidence**, (%)  Pulmonary embolism | *Radiated* | *HL* | 0.0 | 0.6 | 1.2 | 1.2 | 2.9 | 2.9 | † | † |
|  |  | *control* | 0.0 | 0.0 | 0.0 | 0.0 | 0.0 | 0.0 |  |  |
|  | *Radiation-free* | *HL* | 0.0 | 0.8 | 1.3 | 1.6 | 1.8 | 2.6 | † | 5.98 (2.08-17.26),  p<0.001 |
|  |  | *control* | 0.0 | 0.1 | 0.1 | 0.3 | 0.3 | 0.5 |  |  |
| **Cum. incidence,** (%)  Other lung diseases** | *Radiated* | *HL* | 0.0 | 1.7 | 2.7 | 5.5 | 11.5 | 11.5 | 1.10 (0.47-2.62),  p=0.827 | 2.32 (1.10-4.88),  p=0.027 |
|  |  | *control* | 0.0 | 0.6 | 1.8 | 2.3 | 3.1 | 3.1 |  |  |
|  | *Radiation-free* | *HL* | 0.0 | 0.9 | 3.9 | 5.0 | 7.0 | 8.7 | 1.53 (0.85-2.74),  p=0.152 | 2.24 (1.49-3.39),  p<0.001 |
|  |  | *control* | 0.0 | 0.4 | 1.1 | 2.4 | 3.3 | 4.8 |  |  |
| * Acute bronchiolitis ICD-10: J21, bronchitis ICD-10: J20, J40, tuberculosis ICD-10: A15, A16, A19  ** pneumonitis ICD-10: J69, chronic bronchitis ICD-10: J42, hypersensitivity pneumonitis ICD-10: J67, asthma ICD-10: J45, other interstitial pulmonary disease ICD-10: J48, emphysema ICD-10: J43, bronchiectasis ICD-10: J47  † not available due to zero events in cases and/or controls  RR = rate ratio | | | | | | | | | | |

B) in the older (≥50 years) group

|  | **Radiation status** | **Group** | **Follow-up time (years)** | | | | | | **Baseline**  **RR,**  **p-value** | **Follow-up RR,**  **p-value** |
| --- | --- | --- | --- | --- | --- | --- | --- | --- | --- | --- |
|  |  |  | **0** | **1** | **5** | **10** | **15** | **20** |  |  |
| **Cum. incidence,** (%)  Pneumonia | *Radiated* | *HL* | 0.0 | 8.0 | 13.5 | 21.0 | 26.5 | 26.5 | 2.17 (0.83-5.64),  p=0.117 | 1.83 (1.12-2.97),  p=0.017 |
|  |  | *control* | 0.0 | 0.8 | 10.6 | 12.4 | 23.6 | 23.6 |  |  |
|  | *Radiation-free* | *HL* | 0.0 | 13.1 | 21.0 | 25.0 | 29.6 | 34.4 | 4.29 (2.74-6.76),  p<0.001 | 2.77 (2.19-3.51),  p<0.001 |
|  |  | *control* | 0.0 | 1.3 | 7.7 | 13.3 | 18.5 | 24.3 |  |  |
| **Cum. incidence,** (%)  Other lung infections* | *Radiated* | *HL* | 0.0 | 1.6 | 4.6 | 5.6 | 11.6 | 11.6 | † | 3.48 (1.14-10.67),  p=0.029 |
|  |  | *control* | 0.0 | 0.0 | 1.5 | 2.3 | 2.3 | 2.3 |  |  |
|  | *Radiation-free* | *HL* | 0.0 | 2.9 | 5.1 | 5.7 | 7.4 | 8.0 | 2.80 (1.36-5.78),  p=0.005 | 3.56 (2.10-6.04),  p<0.001 |
|  |  | *control* | 0.0 | 0.5 | 1.3 | 2.4 | 4.4 | 4.4 |  |  |
| **Cum. incidence,** (%)  Pulmonary embolism | *Radiated* | *HL* | 0.0 | 2.0 | 3.4 | 4.1 | 4.1 | 4.1 | † | 2.41 (0.74-7.81),  p=0.143 |
|  |  | *control* | 0.0 | 0.4 | 2.0 | 2.0 | 2.0 | 2.0 |  |  |
|  | *Radiation-free* | *HL* | 0.0 | 2.0 | 3.2 | 3.5 | 3.8 | 3.8 | 5.00 (1.11-22.63),  p=0.038 | 1.91 (1.06-3.45),  p=0.029 |
|  |  | *control* | 0.0 | 0.1 | 1.6 | 2.4 | 3.9 | 4.5 |  |  |
| **Cum. incidence,** (%)  Other lung diseases** | *Radiated* | *HL* | 0.0 | 2.4 | 5.5 | 6.2 | 9.8 | 9.8 | 0.80 (0.32-2.02),  p=0.638 | 2.68 (1.04-6.83),  p=0.042 |
|  |  | *control* | 0.0 | 0.0 | 1.1 | 3.7 | 6.3 | 6.3 |  |  |
|  | *Radiation-free* | *HL* | 0.0 | 1.8 | 3.9 | 5.6 | 8.9 | 10.1 | 1.95 (1.13-3.38),  p=0.015 | 1.72 (1.14-2.59),  p=0.010 |
|  |  | *control* | 0.0 | 0.6 | 3.7 | 6.4 | 7.3 | 7.3 |  |  |
| * Acute bronchiolitis ICD-10: J21, bronchitis ICD-10: J20, J40, tuberculosis ICD-10: A15, A16, A19  ** pneumonitis ICD-10: J69, chronic bronchitis ICD-10: J42, hypersensitivity pneumonitis ICD-10: J67, asthma ICD-10: J45, other interstitial pulmonary disease ICD-10: J48, emphysema ICD-10: J43, bronchiectasis ICD-10: J47  † not available due to zero events in cases and/or controls  RR = rate ratio | | | | | | | | | | |
